# Identification and validation of an eight-lncRNA signature that predicts prognosis in patients with esophageal squamous cell carcinoma

**DOI:** 10.1186/s11658-022-00331-x

**Published:** 2022-05-16

**Authors:** Jinfeng Zhang, Xiaodong Ling, Chengyuan Fang, Jianqun Ma

**Affiliations:** Department of Thoracic Surgery, Esophagus and Mediastinum, Harbin Medical University Cancer Hospital, No.150 Hapin Road, Harbin, 150001 Heilongjiang China

**Keywords:** Esophageal squamous cell carcinoma, Long noncoding RNA, Signature, Nomogram

## Abstract

**Background:**

Esophageal squamous cell carcinoma (ESCC) is correlated with worse clinical prognosis and lacks available targeted therapy. Thus, identification of reliable biomarkers is required for the diagnosis and treatment of ESCC.

**Methods:**

We downloaded the GSE53625 dataset as a training dataset to screen differentially expressed RNAs (DERs) with the criterion of false discovery rate (FDR) < 0.05 and |log_2_fold change (FC)| > 1. A support vector machine classifier was used to find the optimal feature gene set that could conclusively distinguish different samples. An eight-lncRNA signature was identified by random survival forest algorithm and multivariate Cox regression analysis. The RNA sequencing data from The Cancer Genome Atlas (TCGA) database were used for external validation. The predictive value of the signature was assessed using Kaplan–Meier test, time-dependent receiver operating characteristic (ROC) curves, and dynamic area under the curve (AUC). Furthermore, a nomogram to predict patients’ 3-year and 5-year prognosis was constructed. CCK-8 assay, flow cytometry, and transwell assay were conducted in ESCC cells.

**Results:**

A total of 1136 DERs, including 689 downregulated mRNAs, 318 upregulated mRNAs, 74 downregulated lncRNAs and 55 upregulated lncRNAs, were obtained in the GES53625 dataset. From the training dataset, we identified an eight-lncRNA signature, (ADAMTS9-AS1, DLX6-AS1, LINC00470, LINC00520, LINC01497, LINC01749, MAMDC2-AS1, and SSTR5-AS1). A nomogram based on the eight-lncRNA signature, age, and pathologic stage was developed and showed good accuracy for predicting 3-year and 5-year survival probability of patients with ESCC. Functionally, knockdown of LINC00470 significantly suppressed cell proliferation, G1/S transition, and migration in two ESCC cell lines (EC9706 and TE-9). Moreover, knockdown of LINC00470 downregulated the protein levels of PCNA, CDK4, and N-cadherin, while upregulating E-cadherin protein level in EC9706 and TE-9 cells.

**Conclusion:**

Our eight-lncRNA signature and nomogram can provide theoretical guidance for further research on the molecular mechanism of ESCC and the screening of molecular markers.

**Supplementary Information:**

The online version contains supplementary material available at 10.1186/s11658-022-00331-x.

## Background

Esophageal cancer (EC) is the seventh most common type of malignancy [1], which is histologically divided into two subtypes: esophageal squamous cell carcinoma (ESCC) and esophageal adenocarcinoma (EAC) [2]. Accounting for > 90% of EC cancers, ESCC is the main EC histologic type, particularly in high-incidence areas of Asia and Africa [2, 3]. Recently, major progress has been made in diagnostic and medical management, especially surgical techniques, chemotherapy, and radiotherapy. Unfortunately, most patients with ESCC have suffered extremely poor outcome mainly due to being diagnosed at advanced stage[4, 5]. Hence, there is an urgent need for identification of reliable biomarkers and targets associated with the prognosis of ESCC.

Nowadays, long noncoding RNAs (lncRNAs) are defined as a class of non-protein-coding RNA transcripts larger than 200 nucleotides in length [6], which have important regulatory roles in multiple biological processes, including cell differentiation, proliferation, glucose metabolism, and immune response [7, 8]. Aberrantly expressed lncRNAs have contributed to the progression of ESCC pathogenesis from the view of prognosis and cellular functions. For example, upregulation of LINC01296 was associated with poor prognosis and promoted cell proliferation and migration in ESCC [9]. Gao et al. [10] highlighted the pivotal role of lncRNA CASC9 as a novel diagnostic, prognostic biomarker, and a potential therapeutic target of ESCC. Similarly, LOC100133669 was upregulated in ESCC tissues, and high LOC100133669 expression was associated with poor prognosis of patients with ESCC [11]. Nevertheless, our knowledge on the prognostic role of lncRNAs in ESCC is far from sufficient. Currently, the advancement of high-throughput microarray platforms has helped us perform comprehensive and systemic analysis of lncRNA profiling analysis in ESCC prognosis.

Two major online databases have provided comprehensive cancer genomic datasets: Gene Expression Omnibus (GEO; http://www.ncbi.nlm.nih.gov/geo/) database, a comprehensive library of gene expression in the National Center of Biotechnology Information (NCBI) [12], and The Cancer Genome Atlas (TCGA, https://gdc-portal.nci.nih.gov/), launched in 2006 by the National Cancer Institute (NCI) and the National Human Genome Research Institute (NHGRI), which contains RNA sequencing (RNA-seq) data and is the database with the most large-scale sequencing results [13]. The methods of mining these two databases mainly focus on the screening of differentially expressed RNAs (DERs) and the analysis of gene regulation networks.

Considering the updated gene expression data and related prognostic information in GEO and TCGA databases, we downloaded lncRNA data, screened DERs, constructed support vector machine (SVM) classifier, and established and validated a risk prediction model for survival prognosis. In addition, we validated the roles of the target gene in vitro.

## Materials and methods

### Dataset preparation

The gene expression profile GSE53625 [14], including 179 ESCC tumor samples and matched controls, was downloaded from Gene Expression Omnibus (GEO: http://www.ncbi.nlm.nih.gov/geo/) database [15] under the GPL18109 platform (Agilent human lncRNA + mRNA array V.2.0). These 179 samples from GEO were used as a training set. Meanwhile, the data of RNA-seq expression, including 161 tumor tissue samples (80 squamous carcinoma and 81 adenocarcinoma) and 11 controls (platform: Illumina HiSeq 2000 RNA Sequencing), were obtained from the TCGA database. We kept 80 squamous carcinoma sample as the validation set. Statistical clinical information of patients in the training set and validation set is summarized in Table 1.

**Table 1:** Clinical characteristics of patients with ESCC in this study

Clinical characteristics	Training set (GSE53625, *N* = 179)	Validation set (TCGA, *N* = 80)
Age (years, mean ± SD)	59.34 ± 9.03	58.19 ± 10.49
Gender (male/female)	146/33	69/11
Alcohol (yes/no/–)	106/73	59/19/2
Tobacco (yes/no)	114/65	42/38
Pathologic N (N0/N1/N2/N3/–)	83/62/22/12	45/26/5/1/3
Pathologic T (T1/T2/T3/T4/–)	12/27/110/30	7/27/41/3/2
Pathologic stage (I/II/III/IV/–)	10/77/92/0	6/47/22/3/2
Arrhythmia (yes/no)	43/136	–
Pneumonia (yes/no)	15/164	–
Anastomotic leak (yes/no)	12/167	–
Adjuvant therapy (yes/no/–)	104/45/30	–
Death (dead/alive)	106/73	25/65
Overall survival time (months, mean ± SD)	36.25 ± 22.86	16.37 ± 12.28

### Identification of significantly DERs

Differential expression analyses were performed for the identification of differentially expressed RNAs (DERs), including lncRNAs and mRNAs (hereafter referred to as “DElncRNAs” and “DEmRNAs,” respectively) between 179 tumor samples and 179 control samples using Limma package version 3.34.7 in R3.4.1 language [16]. The same cutoff value (FDR < 0.05 and |log_2_FC|) was taken as the inclusion criteria for selection of DElncRNAs and DEmRNAs. According to the value of DERs in training set, pheatmap version 1.0.8 in R3.4.1 language [17] based on centered Pearson correlation algorithm [18] was utilized to perform bidirectional hierarchical clustering for describing the gene expression differences between tumor samples and control samples.

### Construction and evaluation of SVM classifier

Combined with survival information in training set, we performed univariate Cox regression analysis from survival package version 2.41–1 in R3.4.1 language [19] to screen significantly prognostic-related DERs (PDERs, including PDElncRNAs and PDEmRNAs) with log-rank *p*-value < 0.05 as the cutoff criterion. The screened PDElncRNAs were used to conduct recursive feature elimination (RFE) analysis in caret package in R3.4.1 language [20, 21] to extract the optimal feature genes with the minimum root mean square error (RMSE) obtained by the 100-fold cross-validation. Subsequently, these optimal feature genes were applied to construct Sigmoid kernel support vector machine (SVM) model using the R3.4.1 e1071 package (https://cran.r-project.org/web/packages/e1071) [22]. We then evaluated the model’s performance in GSE53625 training set and TCGA validation set using area under the curve (AUC) in receiver operating characteristic (ROC) curve. Meanwhile, we calculated each index value of the ROC curve, including sensitivity, specificity, positive prediction value (PPV), and negative prediction value (NPV).

### Identification of signature lncRNAs and RS calculation

On the basis of the optimal feature genes, signature lncRNAs correlated with independent prognosis were identified using a multivariable Cox proportional hazards model implemented with the R3.4.1 survival package version 2.41–1 [19] with log-rank *p*-value < 0.05 as the cutoff criterion. Then, we calculated risk score (RS) following the risk formula: ∑β_lncRNA_ × Exp_lncRNA_, where β_lncRNA_ indicates the coefficient and Exp_lncRNA_ indicates the expression level of signature lncRNA. Afterwards, all patients in training set and validation set were divided into high-risk and low-risk groups according to their median risk score. We used the Kaplan–Meier method in R3.4.1 survival package version 2.41–1 [19] to analyze the overall survival of the two groups and verified the prediction value of the model by plotting ROC curves for the training set and validation set.

### Independent prognosis analysis and nomogram construction

The prognostic value of clinical variables and the RS calculated based on lncRNA signature in training set was initially assessed in univariate Cox proportional hazards regression analyses. Subsequently, each significantly different variable was further evaluated in a multivariate Cox proportional hazards regression analysis. The log-rank *p*-value < 0.05 was served as the cutoff criterion. Furthermore, a nomogram to predict patients’ 3-year and 5-year prognosis was constructed using R3.4.1 rms package version 5.1–2 (https://cran.r-project.org/web/packages/rms/index.html) [23, 24].

### Prediction analysis of signature lncRNA-related genes and functional enrichment

To evaluate the function of signature lncRNAs, we first identified mRNAs significantly related to the signature lncRNAs via calculating the Pearson correlation coefficient (PCC) between 8 signature lncRNAs and 92 PDEmRNAs in the data from the training set using the cor.test function in R3.4.1 language [25]. After screening the connection pairs with RCC > 0.6, signature lncRNA and PDEmRNAs co-expression network was constructed and visualized using Cytoscape version 3.6.1 [26]. Subsequently, these PDEmRNAs in co-expression network were inputted into David website (https://david.ncifcrf.gov) to perform GO biological process and KEGG pathway enrichment analysis, with *p* < 0.05 as the cutoff value.

### Clinical samples and cell lines

The tissue samples used were collected from the Harbin Medical University Cancer Hospital between September 2018 and October 2019, including 15 ESCC tissues and 15 adjacent tissues, all from surgically removed specimens. The study was approved by the ethics committee of the Harbin Medical University Cancer Hospital, and each patient signed a written informed consent form.

Two ESCC cell lines (EC9706 and TE-9) were purchased from the Cell Bank of Type Culture Collection of Chinese Academy of Sciences (Shanghai, China), which were cultured in DMEM with 10% FBS (Gibco, USA) at 37 °C containing 5% CO_2_.

### Cell transfection

For gene knockdown, EC9706 and TE-9 cells were seeded into six-well plates at a density of 3 × 10^5^ cells per well to 80% confluence and transfected with small interfering RNA targeting LINC00470 (si-LINC00470) or negative control (si-NC) generated by GenePharma (Shanghai, China) in accordance with the instructions of Lipofectamine 3000 Reagents (Invitrogen, USA). After 48 h, cells were harvested for further analysis.

### Quantitative real-time PCR analysis

Total RNA was extracted from tissues and cells using TRIzol reagent (TakaRa, Dalian, China), and reverse transcription was performed with PrimeScript RT Reagent Kit with gDNA Eraser (TakaRa, Dalian, China). Quantitative real-time PCR analysis was conducted on LightCycler 480 II Real-Time PCR System (Roche, Basel, Switzerland) using SYBR Premix Ex Taq II (TakaRa). The primers used in our study were as follows: LINC00470, forward 5′-CGTAAGGTGACGAGGAGCTG-3′ and reverse 5′-GGGGAATGGCTTTTGGGTCA-3′; GAPDH forward 5′- GTCAACGGATTTGGTCTGTATT-3′ and reverse 5′- AGTCTTCTGGGTGGCAGTGAT-3′. The relative expression level LINC00470 was calculated using 2^−ΔΔCT^ method and normalized to GAPDH.

### Cell proliferation assay

CCK-8 assay was performed to evaluate the cell proliferation ability in ESCC cells. In brief, transfected cells were inoculated into 96-well plates at a density of 3000 cells per well. At the indicated timepoint (0, 24, 48, and 72 h, respectively), 10 µl of CCK-8 solution (Sigma-Aldrich, USA) was added to each well. After 2 h incubation, the absorbance in each well was measured at 450 nm under a microplate reader.

### Flow cytometry

The cell cycle distribution was analyzed using flow cytometry. Briefly, transfected cells (1 × 10^6^) were harvested, washed with PBS, and fixed by ice-cold ethanol (70%) overnight at 4 °C. Afterwards, cells were washed with PBS twice and stained with propidium iodide (PI) for 30 min at 37 °C. The DNA content of stained cells was determined using BD FACSCalibur flow cytometer (BD Biosciences, Franklin Lakes, NJ, USA) and analyzed with ModFitLT.

### Cell migration assay

Cell migration was measured using transwell 24-well chambers (Corning Inc, Corning, NY, USA). In brief, transfected cells (5 × 10^5^) were harvested and resuspended in serum-free medium. Then, the cell suspensions were added to the upper chamber, and 600 µl medium containing 15% FBS was added to the lower chamber. After 12 h culture, the migratory cells in the lower chamber were fixed with 4% paraformaldehyde for 10 min and stained in 0.5% crystal violet (Sigma-Aldrich, USA) for 30 min. Finally, migratory cells were photographed and counted from five random fields under a light microscope.

### Western blot analysis

Total protein sample was extracted from cell lines with RIPA lysis buffer (Beyotime Institute of Biotechnology, Shanghai, China). Proteins of equal amounts (30 μg) were separated by 10% SDS-PAGE and transferred to PVDF membranes (Millipore). After blocking with 5% nonfat milk, the membranes were incubated with primary antibodies against PCNA (1:1000, ab18197, Abcam), CDK4 (1:1000, ab226474, Abcam), E-cadherin (1:1000, ab219332, Abcam), N-cadherin (1:1000, ab76059, Abcam), and GAPDH (1:5,000; ab8245; Abcam) overnight at 4 °C. After an incubation with horseradish-peroxidase-conjugated secondary antibody (1:5000, SC-2005, Santa Cruz, Inc.) for 2 h at room temperature, the protein bands were visualized with the enhanced chemiluminescence (ECL) Plus kit (Beyotime Institute of Biotechnology).

### Statistical analysis

All quantitative data were analyzed using GraphPad Prism 5 (La Jolla, CA, USA) and expressed as mean ± standard deviation (SD). Differences between si-NC and si-LINC00470 groups were assessed using Student’s *t*-test. A *p*-value of < 0.05 was considered statistically significant.

## Results

### Identification of significantly DERs

**Fig. 1:**
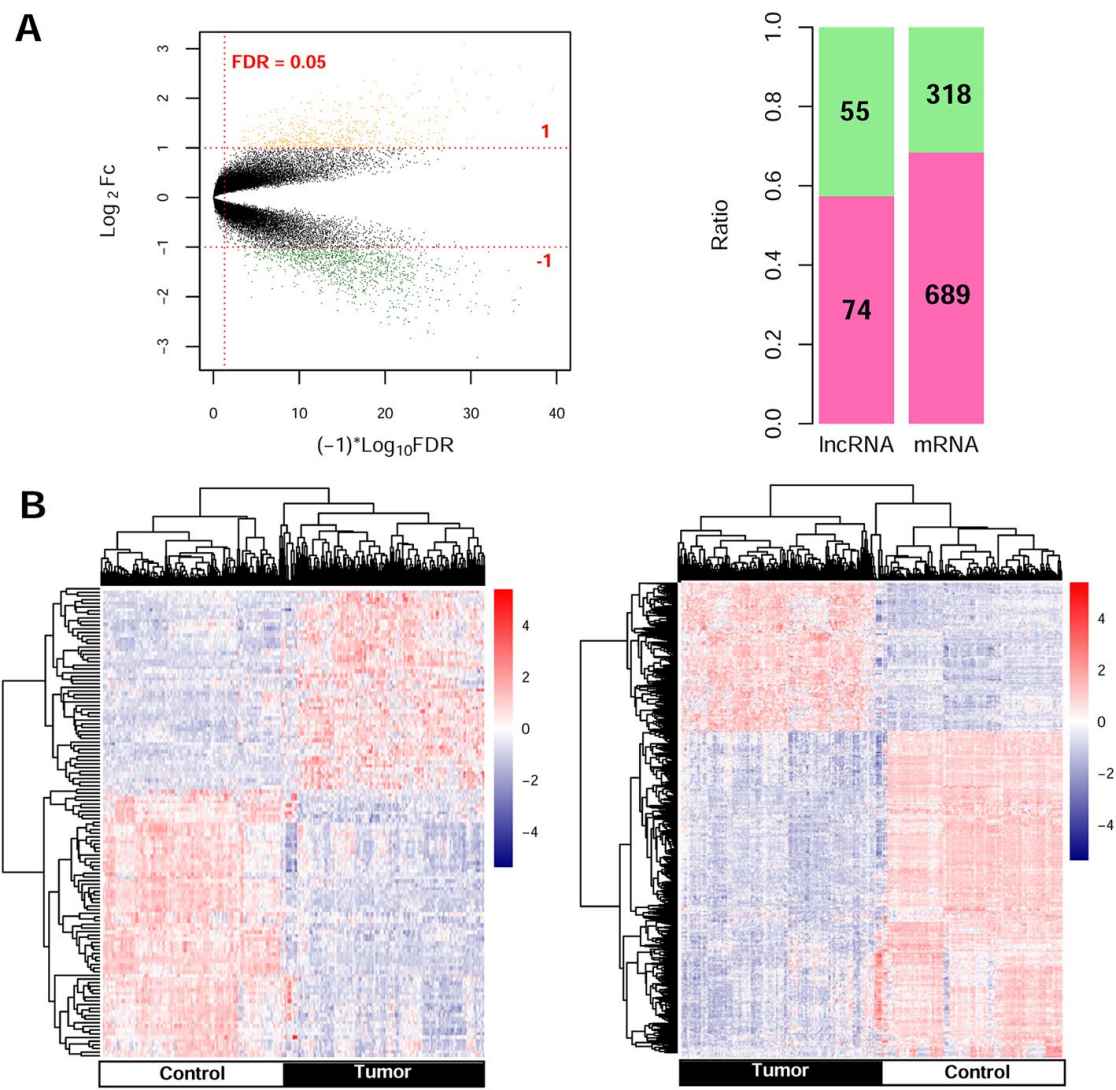
Volcano plot and bidirectional hierarchical clustering heatmap. **A** Left: volcano plot depicting the DEGs; the *X*-axis represents the log-transformed values of false discovery rates, and the *Y*-axis indicates the average differences in gene expression. Green and orange dots indicate the down- and upregulated DEGs in tumor. The red horizontal dotted line indicates FDR < 0.05, and two red vertical dashed lines indicate |log_2_FC|> 1. Right: proportional distribution bar chart of DElncRNAs and DEmRNAs; pink and green represent the significantly upregulated and downregulated percentages of DERs, respectively. **B** Bidirectional hierarchical clustering heat map based on DERs (left lncRNA, right mRNA) expression levels; the white and black samples below represent control and tumor samples, respectively

Significant DERs were first identified among 179 tumor samples compared with 179 control samples in the training set. A total of 129 DElncRNAs (74 downregulated and 55 upregulated) and 1007 DEmRNAs (689 downregulated and 318 upregulated) were identified and are listed in Additional file 1: Table S1. These data were used to build the volcano plot of DElncRNAs and DEmRNAs (Fig. 1A) and the bidirectional hierarchical clustering heatmap (Fig. 1B), indicating the samples tend to cluster in two distinct directions.

### Optimal feature gene selection

**Fig. 2:**
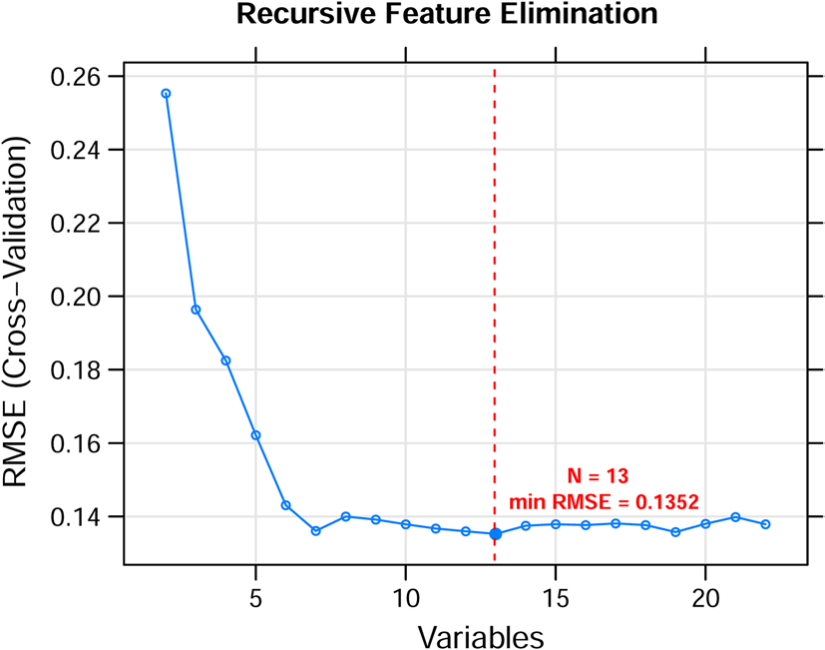
The RMSE curves of the optimal gene combination based on RFE algorithm. The horizontal axis represents the number of lncRNAs variables, and the vertical axis represents cross-validation RMSEs. The marked place is the number of lncRNAs required to obtain the optimal value

**Fig. 3:**
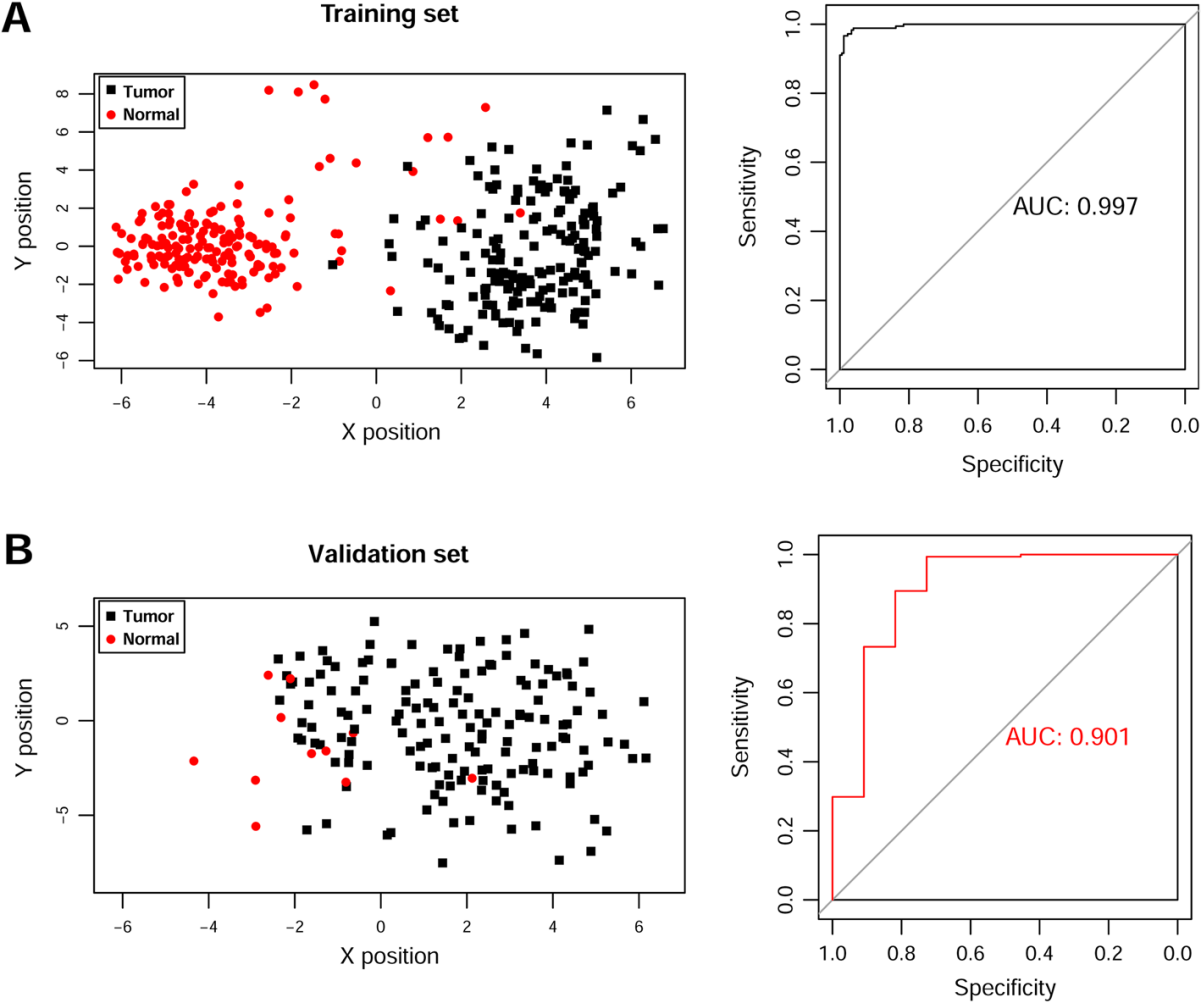
Classification efficiency of the optimum feature genes in the SVM model. The scatter diagram (left picture) and area under the ROC curve (right picture) in the GSE53625 training set **A** and TCGA validation set **B** are shown, respectively. Green dots and red squares represent nonmutated and mutated AML samples, respectively. The *X* and *Y* axes represent the coordinate vector positions of the sample points, respectively

A total of 114 PDERs, including 22 PDElncRNAs and 92 PDEmRNAs, were obtained after univariate Cox regression analysis and are listed in Additional file 2: Table S2. Based on the screened 22 PDElncRNAs, the lncRNA combination with the lowest RMSE was selected as the optimal feature genes in the RFE recursive algorithm screening. As shown in Fig. 2, when the number of lncRNAs was 13, the optimal parameter (minimum RMSE = 0.1352) was obtained, and corresponding 13 optimal feature genes are summarized in Additional file 3: Table S3. A classification model was constructed in training set, whose performance was assessed in the GSE53625 training set and TCGA validation set. The classification results of samples based on the classifier are shown in the scatter diagram in Fig. 3 (left), in which the points with two different colors and shapes are clearly distinguished. The area under the ROC curve is shown in Fig. 3 (right), and corresponding index values of the ROC curve are presented in **Table ****2**. ROC curve analysis revealed an AUC of 0.997 in the training set and 0.901 in the validation set. These results indicate that these optimal feature genes could be used as effective and accurate ESCC diagnostic biomarkers.

**Table 2:** Each index value of the ROC curve in training set and validation set

Datasets	ROC				
	AUC	Sensitivity	Specificity	PPV	NPV
Training set (GSE53625, *N* = 358)	0.997	0.989	0.994	0.994	0.989
Validation set (TCGA, *N* = 173)	0.901	0.933	0.746	0.907	0.909

*AUC* area under the curve, *PPV* positive prediction value, *NPV* negative prediction value

### Identification and validation of an eight-signature lncRNAs

**Fig. 4:**
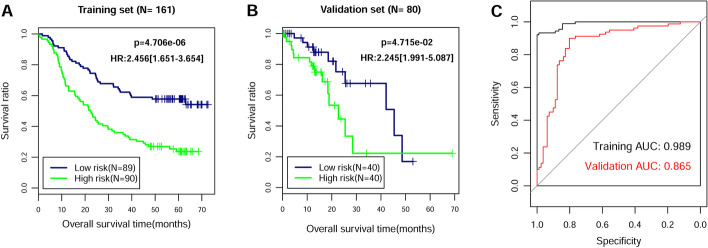
Validation of the eight-lncRNA signature. On the basis of the RS prediction model, prognostic-related Kaplan–Meier curves were drawn in training set (**A**) and validation set (**B**). The blue and green curves represent low- and high-risk group, respectively. **C** The ROC curve of RS prediction model; black and red curves represent the ROC curves of training set and verification set, respectively

Multivariate Cox regression analysis was used to develop signature lncRNAs that are independent predictors of the optimal feature genes in the SVM model. An eight-lncRNA signature was identified, including ADAMTS9-AS1, DLX6-AS1, LINC00470, LINC00520, LINC01497, LINC01749, MAMDC2-AS1, and SSTR5-AS1. The risk coefficients suggested that ADAMTS9-AS1, LINC01497, and MAMDC2-AS1 were risk factors for ESCC (coef > 0), whereas DLX6-AS1, LINC00470, LINC00520, LINC01749, and SSTR5-AS1 appeared to be protective factors (coef < 0) (Table 3). The RS of each patient in the training set and validation set was calculated with the following formula: RS = (0.147172) × Exp_ADAMTS9-AS1_ + (−0.063991) × Exp_DLX6-AS1_ + (−0.112843) × Exp_LINC00470_ + (−0.065239) × Exp_LINC00520_ + (0.184709) × Exp_LINC01497_ + (−0.166036) × Exp_LINC01749_ + (0.104274) × Exp_MAMDC2-AS1_ + (−0.163769) × Exp_SSTR5-AS1_. The higher the risk score, the worse the clinical prognosis. Accordingly, patients were divided into high- and low-risk groups depending on their median risk score to assess the score’s ability to accurately predict survival in a Cox regression model (Additional file 4: Table S4). Kaplan–Meier analysis showed that patients in the low-risk group had better prognosis than those in the high-risk group in the training set (Fig. 4A) and validation set (Fig. 4B). The AUC of the ROC curve was 0.989 in the training set and 0.865 in the validation set (Fig. 4C). These results confirmed that the risk score could be an independent predictor of overall survival.

**Table 3:** An eight-lncRNA signature identified by multivariate Cox regression analysis

ID	Coefficient	*p*-Value	Hazard ratio	95% confidence interval
ADAMTS9-AS1	0.147172	1.641 × 10^−2^	1.159	1.042–1.425
DLX6-AS1	−0.063991	4.324 × 10^−2^	0.938	0.800–0.991
LINC00470	−0.112843	9.950 × 10^−3^	0.893	0.781–0.922
LINC00520	−0.065239	2.393 × 10^−2^	0.937	0.840–0.944
LINC01497	0.184709	1.416 × 10^−2^	1.203	1.004–1.539
LINC01749	−0.166036	4.014 × 10^−2^	0.847	0.575–0.948
MAMDC2-AS1	0.104274	4.851 × 10^−2^	1.110	1.028–1.487
SSTR5-AS1	−0.163769	2.209 × 10^−2^	0.849	0.653–0.903

### The eight-lncRNA signature was an independent predictor of ESCC prognosis

**Fig. 5:**
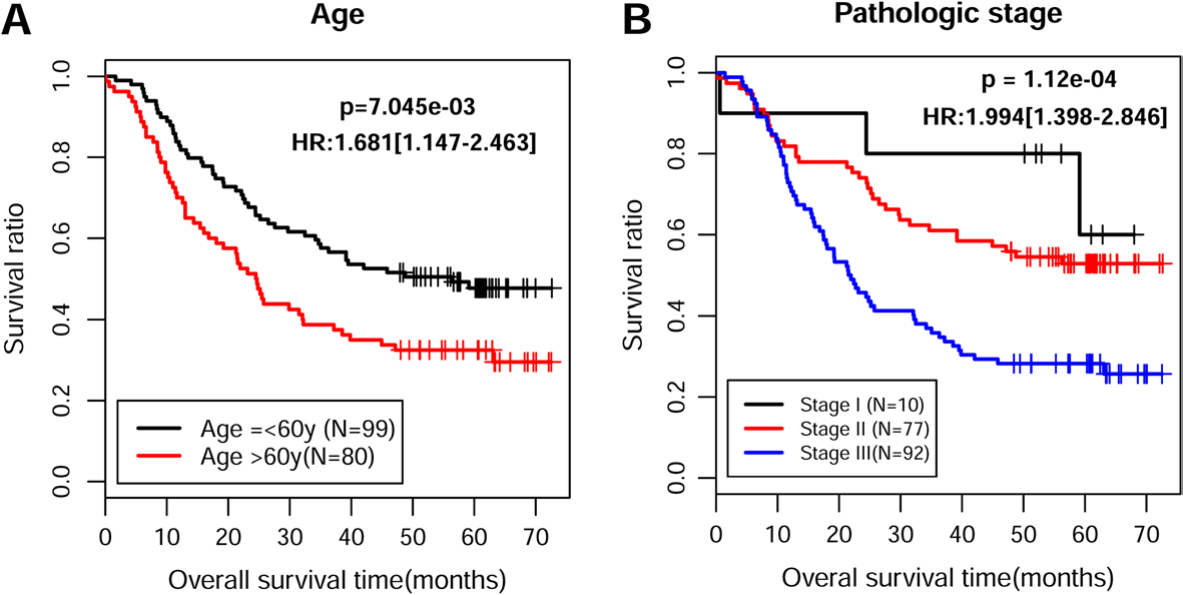
Screening of prognosis-related clinical characteristics by Kaplan–Meier analyses. **A** Kaplan–Meier curves based on different age. The black curve represents patients (≤ 60 years), and red curve represents patients (> 60 years). **B** Kaplan–Meier curves based on different pathologic stages. The black, red, and blue curves represent pathologic I, II, and III sample group, respectively

**Fig. 6:**
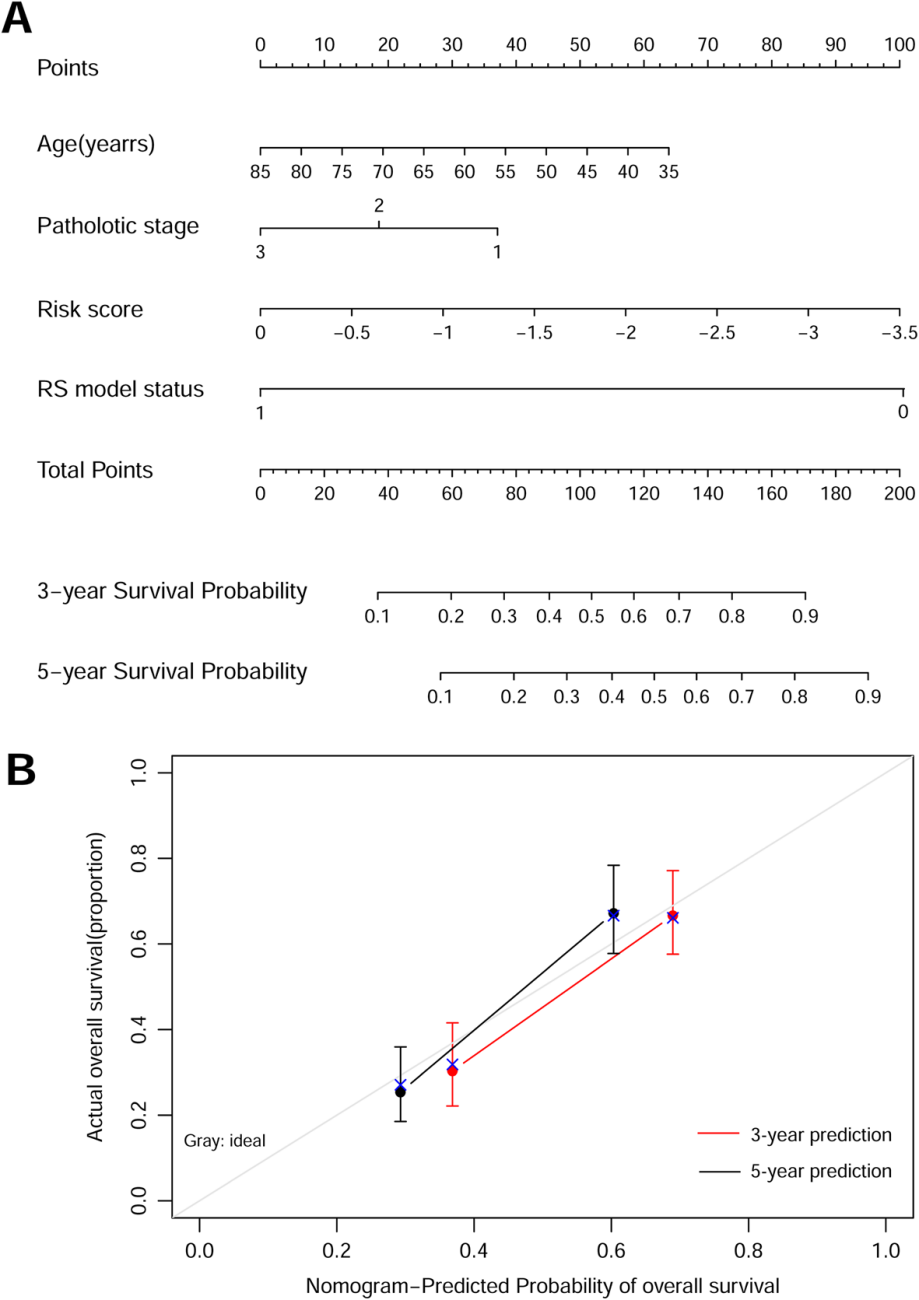
Construction of a nomogram for overall survival prediction in ESCC. **A** Nomogram survival prediction model consists of age, pathologic stage, and RS model status based on the eight-lncRNA signature. **B** A nomogram to predict survival probability at 3 and 5 years after surgery for patients with ESCC, which was compared with actual overall survival in patients with ESCC. The horizontal axis represents the predicted overall survival rate, and the vertical axis represents the actual overall survival rate. The line segments at both ends represent the survival rate obtained in the group with the highest consistency between the predicted and observed values. The red and black lines represent the 3- and 5-year prediction line charts, respectively

To investigate whether the eight-lncRNA signature was an independent predictor of prognosis among patients with ESCC in the training set, we performed univariate and multivariate Cox regression analyses. As illustrated in Table 4, the age, pathologic N, pathologic stage, adjuvant therapy, and RS model status were significantly correlated with patients’ overall survival in the univariate Cox regression. Moreover, the age, pathologic stage, and RS model status based on the eight-lncRNA signature remained three independent predictors. In addition, the results from Kaplan–Meier analysis showed that age (Fig. 5A) and pathologic stage (Fig. 5B) had a significant impact on the prognosis of patients with ESCC (with a log-rank test *p*-value less than 0.0001). Furthermore, a nomogram was constructed that integrated age, pathologic stage, and RS model status to analyze the relationship between these three predictors and survival prognosis (Fig. 6A), which indicated that a higher total number of points on the nomogram presented a worse prognosis. Further analysis suggested that the predicted 3-year and 5-year survival rates by the survival model in the histogram were consistent with the actual 3-year and 5-year survival rates (Fig. 6B).

**Table 4:** Univariate and multivariable Cox proportional-hazards regression analysis on overall survival

	Univariate analysis		Multivariate analysis	
Variables	HR (95% CI)	*p*-Value	HR (95% CI)	*p*-Value
Age (mean ± SD)	1.031 (1.008–1.053)	8.67 × 10^−3^*	1.027 (1.001–1.055)	4.26 × 10^−2^*
Gender (male/female)	0.782 (0.489–1.252)	3.05 × 10^−1^	NA	NA
Alcohol (yes/no)	0.864 (0.588–1.269)	4.55 × 10^−1^	NA	NA
Tobacco (yes/no)	0.749 (0.508–1.105)	1.44 × 10^−1^	NA	NA
Pathologic N (N0/N1/N2/N3)	1.438 (1.181–1.751)	2.51 × 10^−4^*	1.025 (0.751–1.400)	8.75 × 10^−1^
Pathologic T (T1/T2/T3/T4)	1.187 (0.910–1.549)	2.05 × 10^−1^	NA	NA
Pathologic stage (I/II/III/IV)	1.994 (1.398–2.846)	1.12 × 10^−4^*	1.904 (1.062–3.412)	4.58 × 10^−2^*
Arrhythmia (yes/no)	1.120 (0.727–1.725)	6.07 × 10^−1^	NA	NA
Pneumonia (yes/no)	1.425 (0.719–2.823)	3.07 × 10^−1^	NA	NA
Anastomotic leak (yes/no)	1.299 (0.603–2.798)	5.03 × 10^−1^	NA	NA
Adjuvant therapy (yes/no)	2.264 (1.313–3.904)	2.53 × 10^−3^*	1.655 (0.982–2.787)	5.05 × 10^−2^
RS model status (high/low)	2.456 (1.651–3.654)	4.71 × 10^−6^*	2.205 (1.415–3.435)	4.73 × 10^−4^*

*Statistically significant; *RS* risk score, *HR* hazard ratio, *CI* confidence interval, *NA* not analyzed

### Functional characteristics of signature lncRNA-related genes

**Fig. 7:**
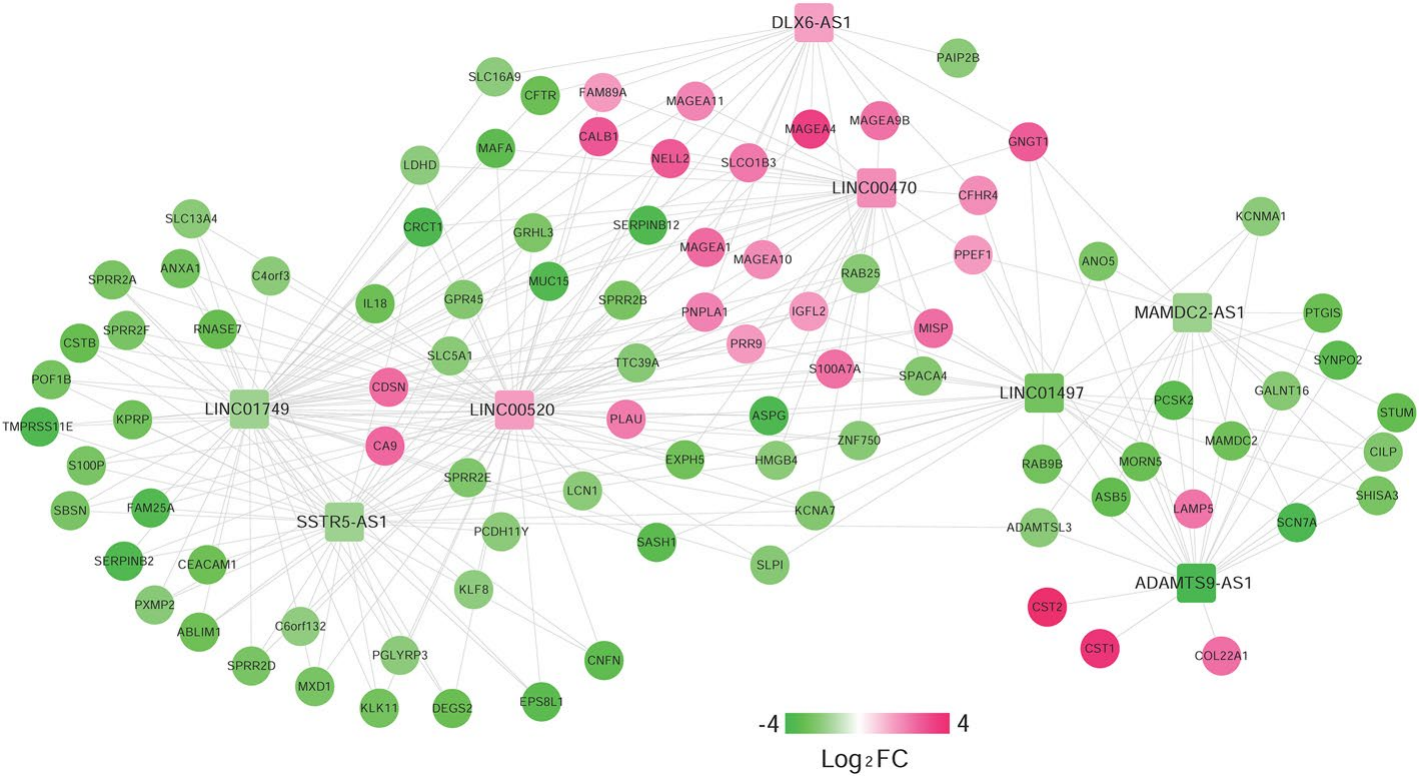
Co-expression network of 8 signature lncRNAs and 74 PDEmRNAs. The change of color from light to dark indicates the change of differential log_2_FC from low to high. Square and circle indicate signature lncRNA and PDEmRNAs, respectively

**Fig. 8:**
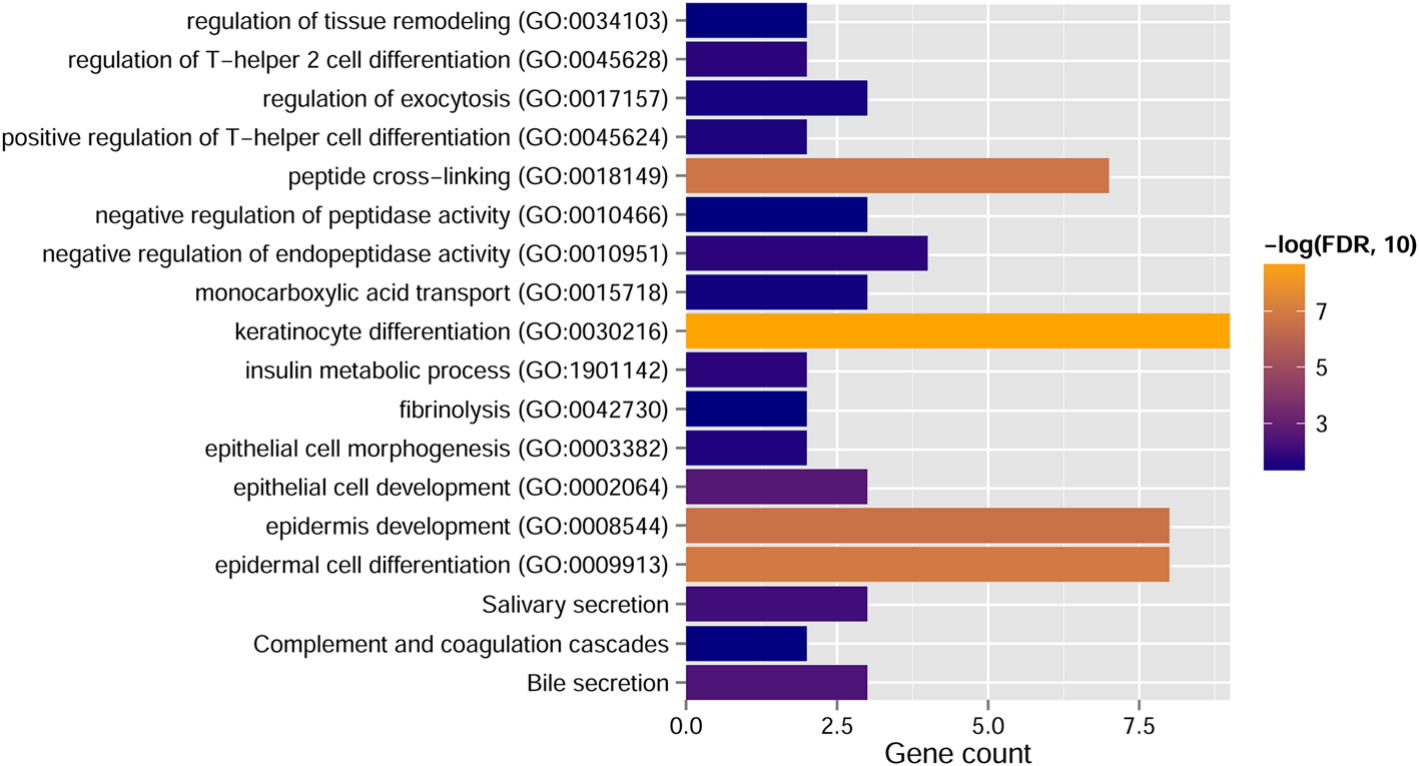
Column diagram of GO and KEGG enrichment analysis. The horizontal axis represents the number of genes, and the vertical axis represents the item name. The color of the column represents the enrichment significance. The closer the color to orange, the higher the significance

We first calculated the PCC between expression levels of 92 PDEmRNAs and eight-lncRNA signature and obtained 279 connection pairs with PCC > 0.6 (Additional file 1: Table S5). A total of 82 nodes, including 8 signature lncRNAs and 74 PDEmRNAs, were obtained in the constructed co-expression network (Fig. 7). Then we performed GO and KEGG functional enrichment analysis for these 74 PDEmRNAs. As shown in Fig. 8 and Table 5, these mRNAs were mainly enriched in the differentiation and development of epidermal and epithelial cells in GO biological process analysis, as well as the secretion of digestive juices in KEGG enrichment analysis.

**Table 5:** Functional annotation of PDEmRNAs in co-expression network

Category	Term	Gene count	*p*-Value	FDR
Biology process	Keratinocyte differentiation (GO:0030216)	9	6.81 × 10^−12^	1.72 × 10^−9^
	Epidermal cell differentiation (GO:0009913)	8	7.94 × 10^−10^	1.00 × 10^−7^
	Peptide cross-linking (GO:0018149)	7	2.02 × 10^−9^	1.70 × 10^−7^
	Epidermis development (GO:0008544)	8	3.63 × 10^−9^	2.29 × 10^−7^
	Epithelial cell development (GO:0002064)	3	5.92 × 10^−5^	2.49 × 10^−3^
	Insulin metabolic process (GO:1901142)	2	7.22 × 10^−4^	2.03 × 10^−2^
	Regulation of T-helper-2 cell differentiation (GO:0045628)	2	7.22 × 10^−4^	2.03 × 10^−2^
	Negative regulation of endopeptidase activity (GO:0010951)	4	5.86 × 10^−4^	2.03 × 10^−2^
	Epithelial cell morphogenesis (GO:0003382)	2	1.10 × 10^−3^	2.77 × 10^−2^
	Positive regulation of T-helper cell differentiation (GO:0045624)	2	1.31 × 10^−3^	3.01 × 10^−2^
	Regulation of exocytosis (GO:0017157)	3	1.71 × 10^−3^	3.61 × 10^−2^
	Monocarboxylic acid transport (GO:0015718)	3	1.91 × 10^−3^	3.71 × 10^−2^
	Fibrinolysis (GO:0042730)	2	2.66 × 10^−3^	4.72 × 10^−2^
KEGG pathway	Regulation of tissue remodeling (GO:0034103)	2	2.99 × 10^−3^	4.72 × 10^−2^
	Negative regulation of peptidase activity (GO:0010466)	3	2.97 × 10^−3^	4.72 × 10^−2^
	Bile secretion	3	5.35 × 10^−5^	4.33 × 10^−3^
	Salivary secretion	3	9.94 × 10^−5^	8.05 × 10^−3^
	Complement and coagulation cascades	2	5.56 × 10^−4^	4.50 × 10^−2^

### Validation of the expression levels of eight-lncRNA signature in ESCC tissues

**Fig. 9:**
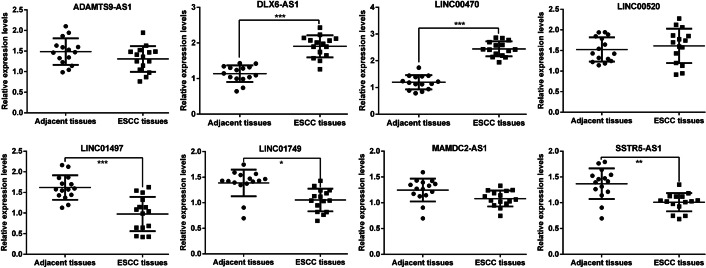
The expression levels of eight signature lncRNAs in ESCC tissues. Quantitative real-time PCR analysis was conducted to determine the expression levels of ADAMTS9-AS1, DLX6-AS1, LINC00470, LINC00520, LINC01497, LINC01749, MAMDC2-AS1, and SSTR5-AS1 in 15 pairs of ESCC tissues and matched adjacent tissues

Quantitative real-time PCR analysis was performed to determine the expression levels of eight-lncRNA signature in 15 pairs of tumor tissues and matched adjacent tissues derived from patients with ESCC. As shown in Fig. 9, the expression levels of DLX6-AS1 and LINC00470 were significantly upregulated, while LINC01479, LINC01749, and SSTR5-AS1 were markedly downregulated in ESCC tissues compared with adjacent tissues. However, there was no significant differences in expression levels of ADAMTS9-AS1, LINC00520, or MAMDC2-AS1 between two groups. According to the higher fold change, we selected LINC00470 for subsequent functional assays.

### Knockdown of LINC00470 suppresses ESCC cell proliferation, G1/S transition, and migration

**Fig. 10:**
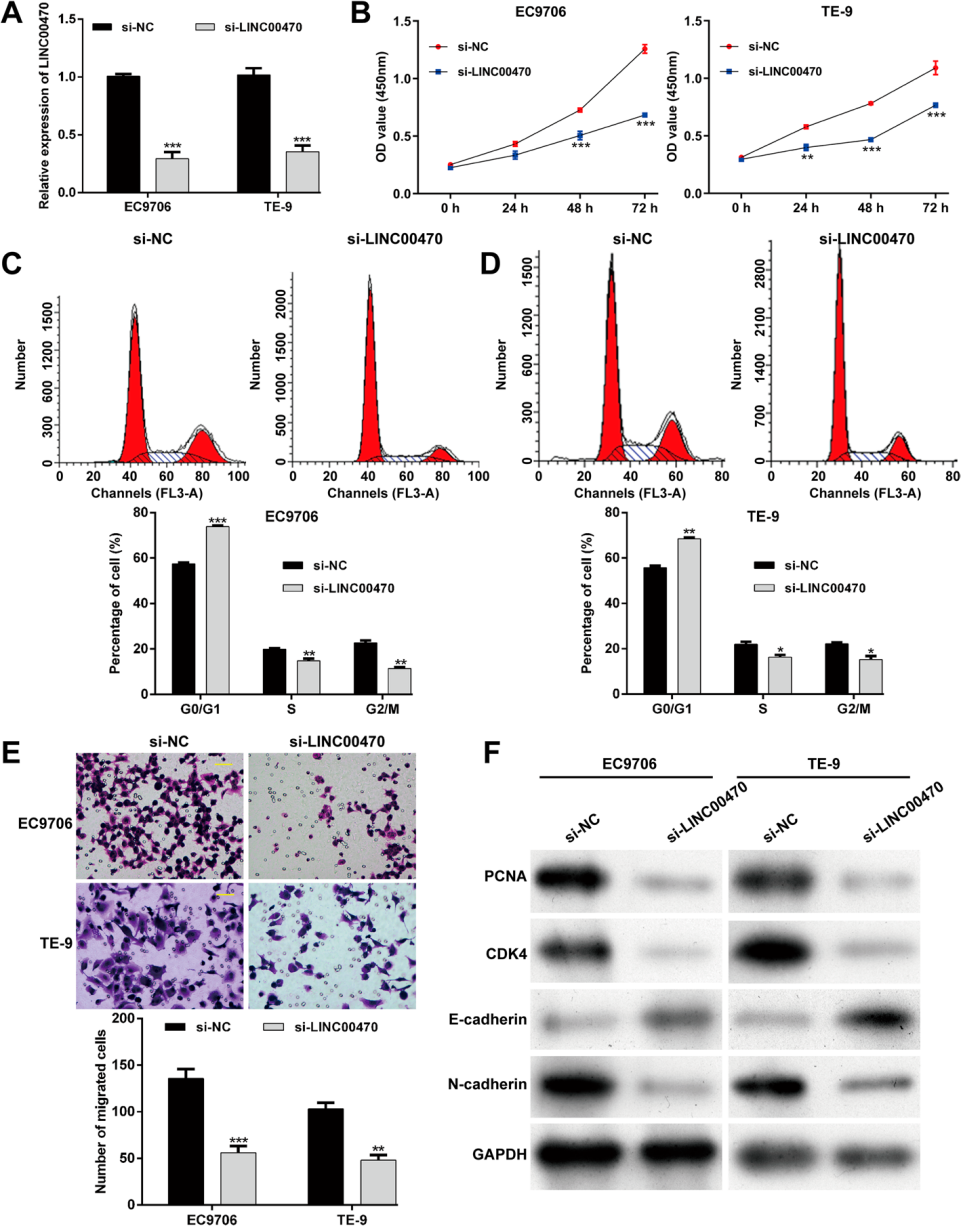
Knockdown of LINC00470 suppresses ESCC cell proliferation, G1/S transition, and migration in vitro. **A** Transfection with si-LINC00470 dramatically suppressed LINC00470 expression in EC9706 and TE-9 cells. **B** CCK-8 assay showed that knockdown of LINC00470 resulted in growth retardation of EC9706 and TE-9 cells. Flow cytometry assay was conducted to analyze cell cycle distribution in transfected EC9706 **C** and TE-9 **D** cells. **E** Cell migration was evaluated in transfected EC9706 and TE-9 cells by transwell assay. Magnification, ×200; scale bar, 100 μm. **F** Western blot analysis was performed to determine the protein levels of PCNA, CDK4, E-cadherin, and N-cadherin in EC9706 and TE-9 cells. Data are expressed as mean ± SD. **p < 0.01, ***p < 0.001, compared with si-NC

To investigate the function of LINC00470 in ESCC in vitro, LINC00470 expression was first knocked down in EC9706 and TE-9 cells by using si-LINC00470 transfection, which was demonstrated by quantitative real-time PCR analysis (Fig. 10A). CCK-8 assay showed that knockdown of LINC00470 resulted in growth retardation of EC9706 and TE-9 cells (Fig. 10B). Moreover, the percentage of cells at G0/G1 phase was significantly increased, in accordance with S and G2/M phase being decreased in si-LINC00470 group compared with si-NC group in both EC9706 (Fig. 10C) and TE-9 (Fig. 10D) cells. In addition, transwell assay indicated that knockdown of LINC00470 markedly inhibited the cell migration ability in EC9706 and TE-9 cells (Fig. 10E). At the molecular level, knockdown of LINC00470 downregulated the protein levels of PCNA, CDK4, and N-cadherin, while upregulating E-cadherin protein level in EC9706 and TE-9 cells (Fig. 10F). The above results demonstrate that knockdown of LINC00470 can inhibit the proliferation and migration of ESCC cells.

## Discussion

To the best of our best knowledge, the tumor–node–metastasis (TNM) staging system acts as the main transitional algorithm to direct the treatment strategies and also serves as a prognostic predictor, but fails to consider the genetic alterations in most types of cancers, including ESCC [27, 28]. In recent years, identification of lincRNA-based signatures has received great attention for its potential to aid in the prognosis of cancers, including hepatocellular carcinoma [29], bladder cancer [30], and pancreatic cancer [31].

In the present study, we first identified 1136 significantly DEGs between tumor tissues and normal tissues in GEO data and confirmed 114 DEGs correlated with prognosis. Finally, eight-lncRNA signature (DLX6-AS1, LINC00470, LINC01479, LINC01749, SSTR5-AS1, ADAMTS9-AS1, LINC00520, and MAMDC2-AS1) was constructed for ESCC. Importantly, a robust nomogram consisting of age, pathologic stage, and RS model status based on the eight-lncRNAs signature was constructed for prediction of prognosis for patients with ESCC. Further analysis suggested the predicted 3-year and 5-year survival rates by the survival model in the histogram were consistent with the actual 3- and 5-year survival rates. By integrating diverse prognostic variables based on clinical characteristics, nomogram has been a widely used tool in oncology that could determine individual probability [32]. Here, our data suggest that our constructed nomogram had better predictive accuracy than each factor alone. Similar to our data, Khalil et al. [33] established a three-lncRNA signature and demonstrated that it could precisely predict overall survival and disease-free survival for ESCC. Three-lncRNA signature (RP11-366H4.1.1, LINC00460, and AC093850.2) was constructed by random forest algorithm and support vector machine algorithm and identified to be potential predictor of overall survival for patients with ESCC [34]. In addition, Mao et al. [32] identified a robust seven-lncRNA signature associated with overall survival that was independent of classical prognostic factors and molecular subtypes in ESCC. The different lncRNA signatures identified in ESCC might be mainly ascribed to different sample resources, sample sizes, and analysis methods. Subsequently, our data showed that 74 PDEmRNAs in co-expression network were mainly enriched in the differentiation and development of epidermal and epithelial cells, as well as the secretion of digestive juices. Consistently, ESCC progression was closely associated with epidermal and epithelial cell differentiation and growth [35, 36].

Subsequently, we confirmed that the expression levels of DLX6-AS1 and LINC00470 were significantly upregulated, while LINC01479, LINC01749, and SSTR5-AS1 were markedly downregulated in ESCC tissues compared with adjacent tissues. By searching published articles, we found that no review had explored the intriguing mechanisms of these five lncRNAs in ESCC, except DLX6-AS1. Several studies have demonstrated that DLX6-AS1 is associated with malignant progression and promotes cell growth and metastasis in ESCC cells [37–39]. Considering the relatively higher increased fold change in expression level, we selected LINC00470 for further functional experiments. As expected, knockdown of LINC00470 significantly suppressed cell proliferation, G1/S transition, and migration in two ESCC cell lines (EC9706 and TE-9). In fact, LINC00470 has been reported to be an oncogene in other malignant tumors. For instance, Wu et al. [40] reported that LINC00470 promoted glioma cell proliferation and invasion and attenuated chemosensitivity. Yan et al. [41] performed overexpression and knockdown experiments to demonstrate the oncogenic functions of LINC00470 on gastric cancer cell proliferation, migration, and invasion. The findings by Huang et al. [42] indicated that knockdown of LINC00470 expression inhibited cell proliferation and cell cycle progression, while overexpression of LINC00470 showed the opposite effects in hepatocellular carcinoma. In addition, LINC00470 promoted invasiveness, migration, and angiogenesis of endometrial cancer cells [43]. Knockdown of LINC00470 could significantly inhibit the melanoma cell proliferation and migration, and suppress the growth of tumor in vivo [44]. On the basis of this evidence, we speculate that high LINC00470 expression appears to be related to poor prognosis in ESCC. It must be mentioned that there are several limitations to this study, including lack of further in vitro experimental study and in vivo data to validate the prognostic performance of our proposed lncRNA signature.

## Conclusion

In summary, our findings identified and validated an eight-lincRNA signature and nomogram as reliable prognostic tools for ESCC. These eight hub genes (ADAMTS9-AS1, DLX6-AS1, LINC00470, LINC00520, LINC01497, LINC01749, MAMDC2-AS1, and SSTR5-AS1) may offer novel therapeutic strategies for patients with ESCC.

## Supplementary Information

**Additional file 1.Table S1**. Identification of DElncRNAs and DEmRNAs.

**Additional file 2.Table S2**. List of total PDERs after univariate cox regression analysis.

**Additional file 3.Table S3**. List of optial feature genes.

**Additional file 4.Table S4**. Summary of patients in high- and low-risk groups.

**Additional file 5.Table S5**. List of lncRNA signature and corresponding connection pairs.

## Data Availability

All datasets generated for this study are included in the manuscript.

## Abbreviations

ESCC: Esophageal squamous cell carcinoma
RS: Risk score
EC: Esophageal cancer
NCBI: National Center of Biotechnology Information
NCI: National Cancer Institute
RNA-Seq: RNA sequencing
DEGs: Differentially expressed genes
SVM: Support vector machine
TNM: Tumor–node–metastasis
